# The driving force for co-translational protein folding is weaker in the ribosome vestibule due to greater water ordering†

**DOI:** 10.1039/d1sc01008e

**Published:** 2021-08-03

**Authors:** Quyen V. Vu, Yang Jiang, Mai Suan Li, Edward P. O'Brien

**Affiliations:** Institute of Physics, Polish Academy of Sciences Al. Lotnikow 32/46 02-668 Warsaw Poland masli@ifpan.edu.pl; Department of Chemistry, Penn State University University Park Pennsylvania USA epo2@psu.edu; Institute for Computational Sciences and Technology Quang Trung Software City, Tan Chanh Hiep Ward, District 12 Ho Chi Minh City Vietnam; Bioinformatics and Genomics Graduate Program, The Huck Institutes of the Life Sciences, Penn State University University Park Pennsylvania USA; Institute for Computational and Data Sciences, Penn State University University Park Pennsylvania USA

## Abstract

Interactions between the ribosome and nascent chain can destabilize folded domains in the ribosome exit tunnel's vestibule, the last 3 nm of the exit tunnel where tertiary folding can occur. Here, we test if a contribution to this destabilization is a weakening of hydrophobic association, the driving force for protein folding. Using all-atom molecular dynamics simulations, we calculate the potential-of-mean force between two methane molecules along the center line of the ribosome exit tunnel and in bulk solution. Associated methanes, we find, are half as stable in the ribosome's vestibule as compared to bulk solution, demonstrating that the hydrophobic effect is weakened by the presence of the ribosome. This decreased stability arises from a decrease in the amount of water entropy gained upon the association of the methanes. And this decreased entropy gain originates from water molecules being more ordered in the vestibule as compared to bulk solution. Therefore, the hydrophobic effect is weaker in the vestibule because waters released from the first solvation shell of methanes upon association do not gain as much entropy in the vestibule as they do upon release in bulk solution. These findings mean that nascent proteins pass through a ribosome vestibule environment that can destabilize folded structures, which has the potential to influence co-translational protein folding pathways, energetics, and kinetics.

## Abstract 2

In the ribosome vestibule, the contact minimum between two methane molecules is half as stable as compared to in bulk solution, demonstrating that the hydrophobic effect is weakened in the vestibule of ribosome exit tunnel.

## Graphical abstract

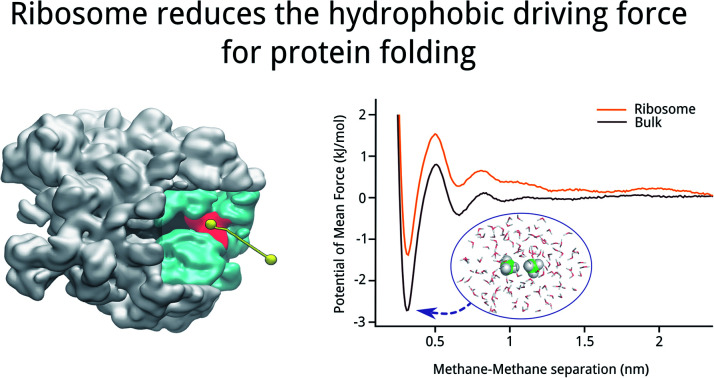

## Introduction

**Fig. 1:**
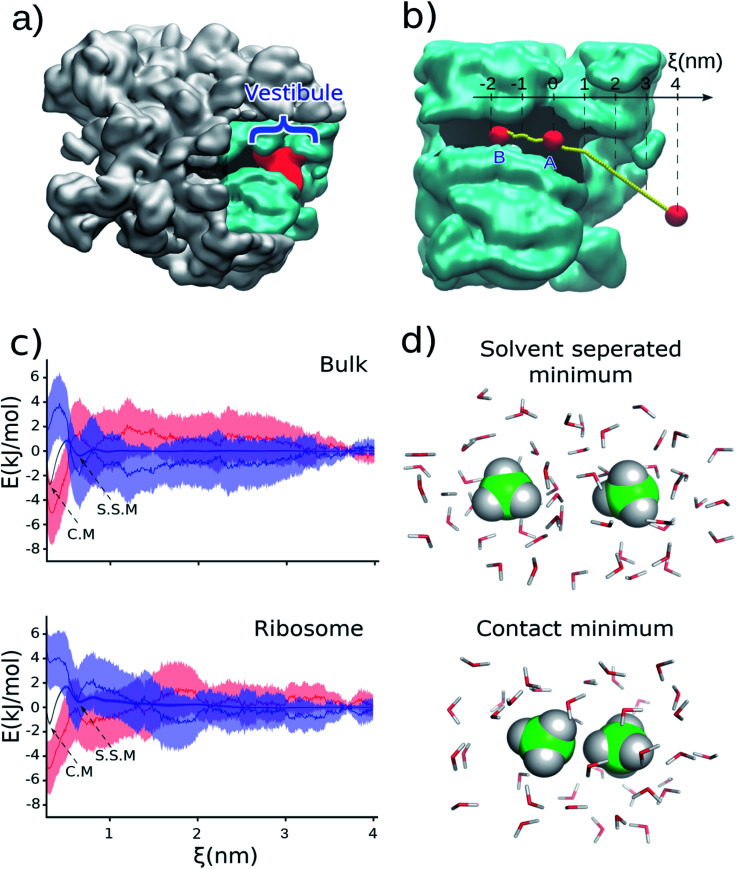
(a) Cross-section of the 50S subunit of *E. coli* highlighting the ribosome (gray), exit tunnel (black), and last 3 nm of the exit tunnel known as the ‘vestibule’ (red) where tertiary folding can occur. (b) The portion of the ribosome exit tunnel used in the simulations. The center-line of the exit tunnel is represented as a yellow dotted line. (c) Potential of mean force (Δ*G*, black), enthalpy (Δ*H*, blue), and negative entropy term (−*T*Δ*S*, red) in bulk (upper) and in the ribosome exit tunnel (lower) between two methanes with one methane at point *A*. The shaded regions present 95% confident intervals calculated from bootstrapping. (d) A snapshot of methanes in solvent-separated minimum and in contact minimum configurations.

The association of hydrophobic side chains is the primary driving force for protein folding.^1,2^ The first location that tertiary protein folding can occur is in the ribosome vestibule, corresponding to the last 3 nm of the ribosome exit tunnel (red region in Fig. 1a). The nascent polypeptide chain passes through the 10 nm exit tunnel that is lined with ribosomal proteins and RNA, and out into the cellular milieu. Simulations first predicted,^3,4^ and experiments later verified,^5,6^ that many domains are sterically permitted to fold in the ribosome vestibule because the vestibule is wider than the rest of the tunnel (diameter is about 3 nm as compared to 1.5 nm).

A variety of changes in folding thermodynamics and kinetics occur as a domain passes through the vestibule and outside the exit tunnel, and some can be protein specific. While co-translational folding can occur in the vestibule according to computer simulations^4^ and cryo-EM structures,^7^ NMR experiments,^8^ and fraction-full-length protein profiles, which are proportional to force,^9^ have found that, with the exception of ADR1 protein,^10^ individual domains in the vestibule are often less stable as compared to the same domain outside the exit tunnel when measured on translationally arrested ribosomes. Even just outside the vestibule the native state is often less stable than in bulk solution.^11,12^

In terms of kinetics, single molecule laser optical tweezer experiments^13–15^ have found that the folding process for two different proteins on stalled ribosomes becomes slower the closer the domain is to the ribosome's outer surface, with the trend line suggesting folding is slower still in the vestibule. Indeed, a number of computer simulations of co-translational folding find slower folding rates near the outer ribosome surface and in the vestibule.^4,16^ Additionally, increasing the salt concentration in solution was found to accelerate domain folding just outside the vestibule indicating electrostatic interactions play a role in this slowdown.^13^ Changes in salt concentration can also change the strength of the hydrophobic effect,^17^ with higher salt concentration leading to increased water density around the component ions compared to pure water.^18^ This suggests the possibility that variation in the hydrophobic effect could arise in the exit tunnel vestibule due to the electrostatic environment it creates.

In this study, we examine whether there is a decrease in the affinity of hydrophobes for one another – a classic measure of the strength of the hydrophobic effect – in the ribosome's vestibule.

To test this hypothesis we carried out classical, all-atom molecular dynamics simulations of the association of two hydrophobic molecules both in the presence and absence of the *Escherichia coli* ribosome at 310 K, the optimal growth temperature of this organism. We calculate the potential of mean force between two methanes (CH_4_) along the center line of the ribosome exit tunnel (Fig. 1b). We study methane because it is a model compound for the alanine side chain. Methanes are also closely chemically related to methyl moieties (CH_2_) – the most common building block for more complex hydrophobic molecules. The transfer free energy of hydrophobic molecules is directly proportional to the number of methyl's, suggesting our results for methane will be relevant to larger hydrophobic side chains. Additionally, the small number of degrees of freedom of methane means that we can obtain precise statistics in the simulations. The center line is the line along the exit tunnel that is maximally separated from all ribosomal atoms (yellow line in Fig. 1b). Since the hydrophobic effect is water mediated, we calculate association along this center line so that the methanes are always solvated (see radial distribution functions in Fig. S1†), and do not come into direct contact with the exit tunnel walls.

## Results and discussion

### Ribosome reduces the hydrophobic driving force for protein folding

Holding one methane fixed at position A (labelled in Fig. 1b), which is about 2.5 nm into the exit tunnel, we find that the potential of mean force between this methane and a methane brought along the center line exhibits a solvent separated minimum (labeled ‘S.S.M.’ in Fig. 1c) and contact minimum (labeled ‘C.M.’ in Fig. 1c). Since the ribosome exit tunnel is 1.5 nm in diameter, on average, we focus on testing for changes in the hydrophobic effect at distances less than this. Therefore, we examine free energy differences between the contact minimum and solvent-separated minimum. We find the contact minimum is 1.74 kJ mol^−1^ (95% confidence interval (CI): [1.70, 1.78] kJ mol^−1^, calculated from bootstrapping) more stable than the solvent separated minimum (Table 1). Carrying out this simulation in bulk solution (*i.e.*, without the ribosome) along the same spatial path shown in Fig. 1b, the contact minimum is 2.31 kJ mol^−1^ (95% CI: [2.26, 2.37], bootstrapping) more stable than the solvent separated minimum (Table 1). Thus, the presence of the ribosome vestibule decreases the stability of the associated methanes (*p*-value < 1 × 10^−6^, one-sided permutation test).

**Table 1:** Free energy, enthalpy, and entropy at contact minimum and solvent separated minimum (95% confidence interval about the mean is reported in parentheses)

System		Contact minimum (kJ mol^−1^)			Solvent-separated minimum (kJ mol^−1^)			Contact minimum minus solvent-separated minimum (kJ mol^−1^)		
		Δ*G*	Δ*H*	*T*Δ*S*	Δ*G*	Δ*H*	*T*Δ*S*	ΔΔ*G*	ΔΔ*H*	*T*ΔΔ*S*
Point *A*	Bulk	−2.71 (−2.82, −2.61)	2.09 (−0.51, 4.72)	4.80 (2.20, 7.42)	−0.40 (−0.49, −0.29)	−1.72 (−4.56, 1.25)	−1.32 (−4.12, 1.58)	−2.31 (−2.37, −2.26)	3.81 (2.88, 4.77)	6.12 (5.23, 7.07)
	Ribosome	−1.31 (−1.47, −1.17)	3.58 (1.32, 5.78)	4.90 (2.63, 7.07)	0.43 (0.30, 0.56)	1.05 (−1.45, 3.50)	0.62 (−1.78, 3.13)	−1.74 (−1.78, −1.70)	2.54 (1.09, 3.81)	4.28 (2.83, 5.55)
Point *B*	Bulk	−3.40 (−3.60, −3.10)	—	—	−0.37 (−0.49, −0.18)	—	—	−3.03 (−3.16, −2.82)	—	—
	Ribosome	−2.35 (−3.07, −1.82)	—	—	0.09 (−0.31, 0.45)	—	—	−2.44 (−3.05, −2.01)	—	—

To test if this conclusion is robust at different positions along the center line of the tunnel, we carried out the same simulations and analyses but with the methanes associated approximately 2 nm further inside the exit tunnel (point B in Fig. 1b – lower region of the ribosome exit tunnel, where helix formation has been experimentally observed to occur). We find that although the relative stabilities are different as compared to point A (Table 1 and Fig. S2 in ESI Text†), which is to be expected in the heterogeneous environment along the exit tunnel, it is still the case that the presence of the ribosome leads to a less stable associated state relative to the solvent separated minimum (−2.44 kJ mol^−1^, 95% CI [−3.05, −2.01] in the ribosome *versus* −3.03 kJ mol^−1^, 95% CI [−3.16, −2.82] in bulk). These results are in agreement with an earlier study on cylindrical confinement.^19^ We conclude from these data that the presence of the ribosome decreases the affinity of hydrophobic molecules for one another in the exit tunnel where co-translational tertiary protein folding can occur.

We note that because we are projecting the non-linear path of the methane (yellow line in Fig. 1b) onto the linear reaction coordinate *ξ* (black line in Fig. 1b) this leads to the situation that in bulk solution the difference in contact *versus* solvent separated minimum stabilities depend on whether they are computed using the path to point *A* or *B* (Fig. 1b and Table 1). The difference in the curvature of the path near point *A* and *B* leads to the projection of different probability densities onto to *ξ*. This is acceptable, however, because we are only interpreting the thermodynamic properties along the same path – *e.g.*, association at point *A* in bulk *versus* association at point *A* in the ribosome exit tunnel.

To understand why the hydrophobic effect is weakened by the ribosome we calculated the entropy and enthalpy of association at position *A* at 310 K using data from multiple temperatures (see Methods†). We find that upon going from the solvent-separated minimum to the contact minimum there is no statistically significant difference in the enthalpy of association (ΔΔ*H*) calculated in bulk solution (3.81 kJ mol^−1^, 95% CI: [2.88, 4.77]) *versus* in the presence of the ribosome (2.54 kJ mol^−1^, 95% CI: [1.09, 3.81], Table 1). The *p*-value is 0.08 (one-sided permutation test) for the difference between these two values. Thus, changes in enthalpy of association do not cause the change in stability of hydrophobic association in the vestibule. We do find a difference, however, in the entropy of association. In bulk solution the entropic term (*T*ΔΔ*S*) is 6.12 kJ mol^−1^ (95% CI [5.23, 7.07]), while on the ribosome it is 4.28 kJ mol^−1^ (95% CI [2.83, 5.55]). The difference between these two values is statistically significant (*p*-value = 0.02, one-sided permutation test). Both entropy terms are positive, meaning that there is a gain in entropy upon association of the two methanes. However, the gain in entropy is smaller in the presence of the ribosome (4.28 kJ mol^−1^*versus* 6.12 kJ mol^−1^). Thus, the ribosome-induced weakening of hydrophobic association arises from a smaller gain in entropy upon going from the solvent separated configuration of the methanes to the associated state.

### The ribosome exit tunnel has more ordered solvent compared to bulk solution

Previous studies have demonstrated that the entropy gain upon the association of hydrophobes arises from the release of several ordered water molecules from the first solvation shell of the methanes, and their subsequent gain in rotational and translational entropy.^20^ This suggests the hypothesis that water molecules are more ordered in the exit tunnel as compared to bulk solution, resulting in a smaller gain in entropy when waters are released upon methane association. Indeed, an earlier simulation study observed greater water ordering in the exit tunnel and reduced rotational entropy.^21^ We tested this hypothesis in two ways. First, we computed the entropy of water in the region around positions *A* and *B* in the absence of methanes, as well as in bulk, using the two-phase thermodynamic method^22–24^ (see Methods in ESI Text†). We find the total entropy of water decreases from 69.19 J K^−1^ mol^−1^ (95% CI: [68.75, 69.46]) in bulk solution, to the smaller values of 61.02 (95% CI: [60.18, 62.37]) and 57.22 (95% CI: [56.01, 58.26]) J K^−1^ mol^−1^, respectively, at points *A* and *B* (see Table 2). And that this decrease arises from a large decrease in water's translational entropy and a smaller decrease in waters rotational entropy (Table 2). (Note well, since the TIP3P water molecules in our simulations are rigid the vibrational entropy is zero and not reported in Table 2.) Thus, water molecules have less translational and rotational entropy in the vestibule.

**Table 2:** Translational, rotational, and total entropy of water in different regions of exit tunnel (95% confidence interval about the mean is reported in parentheses)

Property	System		
	Bulk	Point *A*	Point *B*
Translational entropy (J K^−1^ mol^−1^)	56.04 (55.72, 56.22)	47.89 (47.42, 48.48)	44.53 (43.08, 45.64)
Rotational entropy (J K^−1^ mol^−1^)	13.15 (13.03, 13.24)	13.13 (12.40, 13.89)	12.69 (12.52, 12.93)
Total entropy (J K^−1^ mol^−1^)	69.19 (68.75, 69.46)	61.02 (60.18, 62.37)	57.22 (56.01, 58.26)

**Fig. 2:**
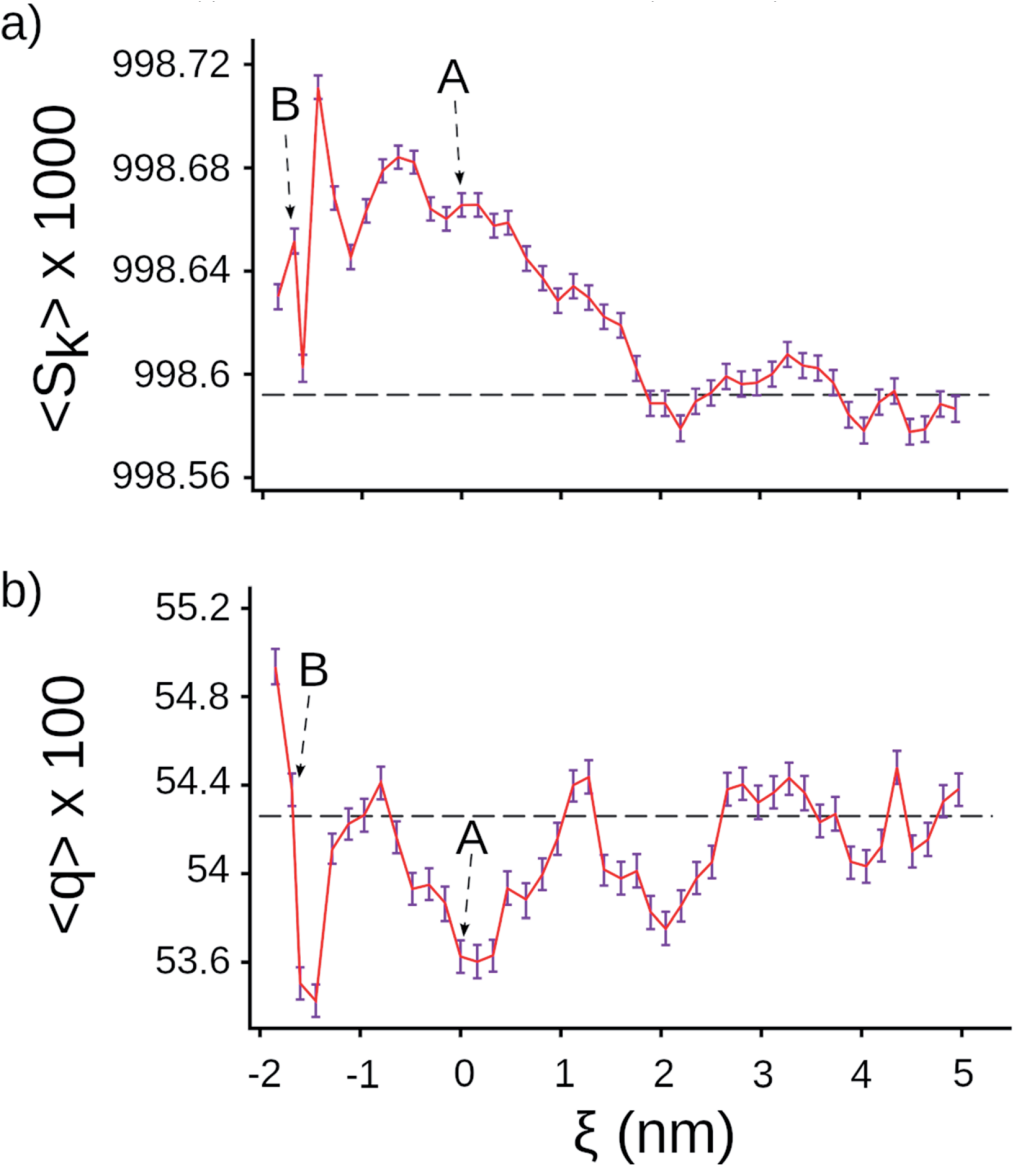
Tetrahedral parameters for water molecules along the center line of the ribosome. (a) Distance order parameter *S*_k_ (eqn (S4)†), (b) orientational order parameter *q* (eqn (S5)†). The horizontal lines are the average value calculated for water molecules in bulk, the error bar presents 95% confident intervals calculated from bootstrapping.

Next, we tested whether we could detect signatures of greater water ordering in the exit tunnel by using the tetrahedral orientational (*q*) and translational (*S*_k_) order parameters.^25–27^ These two metrics measure two different aspects of how closely five water molecules are to forming a tetrahedron, which is the minimum potential energy structure. We computed *q* and *S*_k_ at each point along the center line by selecting the water molecule that was closest to that point and its four nearest-neighbor water molecules (see Methods in ESI Text†). We find that *S*_k_ is higher in the exit tunnel than in bulk (Fig. 2a), indicating that the water molecules adopt a more tetrahedral structure in terms of their distances from the central water molecule. The orientational parameter *q*, however, fluctuates above and below the bulk value, indicating the ribosome distorts the water cluster angular configuration to be more or less tetrahedral at different points along the tunnel (Fig. 2b). The angular degrees-of-freedom of the tetrahedron are softer than the distance degrees-of-freedom, meaning that it takes more energy to change the distances than the angles. Taken together, these results demonstrate that water molecules are more ordered in the exit tunnel and have decreased entropy. They also indicate that the smaller entropy gain upon association of methanes arises from the fact that the newly liberated waters are released into an environment where the water molecules are more ordered and have less entropy.

### Ribosome sites that could influence the hydrophobic effect

Negatively charged residues and groups, such as the phosphates of RNA, lead to more water ordering than positively charged residues, while polar and non-polar residues cause the least changes in water structure.^28^ For small hydrophobic residues water's tetrahedral structure in the first solvation shell is equivalent to bulk water.^29^ Thus, greater water ordering in the exit tunnel is due in part to charged amino acids and the phosphate groups of 23S RNA. For completeness, we have identified all charged and polar residues within 1.5 nm of points *A* and *B* and report them in Table 3.

**Table 3:** Distance between methane molecules at the contact minimum at points *A* and *B*(Fig. 1b) and residues lining the ribosome exit tunnel. Residue indices are followed PDBID: 3R8T

Index	Point *A*			Point *B*		
	Residue	Chain	Distance (Å)	Residue	Chain	Distance (Å)
1	A507	A (23S)	11.4	LYS83	S (L22)	6.77
2	A508	A (23S)	12.0	A471	A (23S)	7.95
3	C1335	A (23S)	12.0	GLN72	T (L23)	8.55
4	G1334	A (23S)	13.0	ARG84	S (L22)	10.0
5	HIS70	T (L23)	13.1	A472	A (23S)	10.1
6	A1322	A (23S)	13.6	A470	A (23S)	10.7
7	A492	A (23S)	13.9	G1259	A (23S)	12.4
8	A91	A (23S)	14.1	C461	A (23S)	12.6
9	C1319	A (23S)	14.4	A1322	A (23S)	13.2
10	U92	A (23S)	14.4	A508	A (23S)	14.7
11	A1336	A (23S)	15.0	U1258	A (23S)	14.9

### Estimated effect on co-translational protein folding

We can estimate how much this weakening of the hydrophobic effect will affect the stability of a typical protein domain. We first note that the stability of the contact minimum is half of its bulk value in the exit tunnel (−1.31 kJ mol^−1^ 95% CI [−1.47, −1.17] *versus* −2.71 kJ mol^−1^ 95% CI [−2.82, −2.61]). Next, we note that the hydrophobic effect contributes 60%, on average, to the free energy difference between the folded and unfolded states.^30,31^ Therefore, the weakening of the hydrophobic effect will decrease the folded state stability by around 30% (=60%/2). A typical 80 residue protein (which can fold in the ribosome vestibule^3,6^) has a free energy of stability of −25 kJ mol^−1^ in bulk solution.^32^ Hence, the stability of folded state is decreased by around −7.5 kJ mol^−1^ (=−25 kJ mol^−1^ × 0.5 × 0.6) due to the reduction of the hydrophobic effect in the exit tunnel. While a rough estimate, it suggests that destabilization on the order of 10's of kJ mol^−1^ is possible.

## Conclusions

In summary, the hydrophobic effect is weaker in the ribosome vestibule because water molecules are more ordered than in bulk. This greater ordering decreases the entropy gain of water molecules released from the first hydration shell of hydrophobic moieties, thereby weakening the entropic driving force for hydrophobic association.

More broadly, understanding the folding of proteins at their earliest stage of existence, *i.e.*, during synthesis, is an area of intense research efforts because what happens during this crucial period can influence the fate of a protein in a cell.^33^ Studies have found that for some proteins their folding pathways can differ from that of bulk solution^34–36^ due to the N- to C-terminal synthesis of proteins, interactions between the nascent chain and ribosome,^11,13^ and the speed of protein synthesis.^37^ To this list, our results indicate that the weakening of the hydrophobic effect – the primary driving force of protein folding – is also likely to influence nascent protein folding. The hydrophobic effect is still present in the vestibule, and hence our results are consistent with observations that protein domains do fold in the exit tunnel. However, our results indicate the structures that do form will not be as thermodynamically stable as in bulk solution. This decreased stability in the vestibule has the potential to slow down the rate of protein co-translational folding, and may be a mechanism contributing to slower folding and decreased stability.

In addition, post-translation protein folding may be aided by the presence of the ribosome^38–40^ potentially through outer ribosome surface interactions that are not accessible to nascent chains emerging from the exit tunnel. All of this points to a rich set of scenarios of the role of the ribosome on co- and post-translational protein folding, and the role of solvent in mediating nascent protein behavior.

## Data availability

All the data that support the findings of this study are available from the corresponding authors upon reasonable request.

## Author contributions

E. P. O. designed the research. Q. V. V. and Y. J. conducted the simulations. All authors analyzed the results, wrote and reviewed the article.

## Conflicts of interest

There are no conflicts to declare.

## Supplementary Material

SC-012-D1SC01008E-s001
