# Supplementary material for: The driving force for co-translational protein folding is weaker in the ribosome vestibule due to greater water ordering
Source: Chem Sci. 2021 Aug 3;12(35):11851–7. doi: 10.1039/d1sc01008e (PMC8442680; doi:10.1039/d1sc01008e)
Supplement: SC-012-D1SC01008E-s001 [file SC-012-D1SC01008E-s001.pdf]

Electronic Supplementary Material (ESI) for Chemical Science.
This journal is © The Royal Society of Chemistry 2021

Supplementary Information

The Driving Force for Co-translational Protein Folding is Weaker In the Ribosome Vestibule due to Greater Water Ordering

Quyen V. Vu,[a‡] Yang Jiang,[b‡], Mai Suan Li[ac] and Edward P. O'Brien[bde]

[a]Institute of Physics, Polish Academy of Sciences, Al. Lotnikow 32/46, 02-668 Warsaw, Poland

[b]Department of Chemistry, Penn State University, University Park, Pennsylvania, United States

[c]Institute for Computational Sciences and Technology, Ho Chi Minh City, Vietnam

[d]Bioinformatics and Genomics Graduate Program, The Huck Institutes of the Life Sciences, Penn State University, University Park, Pennsylvania, United States

[e]Institute for Computational and Data Sciences, Penn State University, University Park, Pennsylvania, United States

[‡] These authors contributed equally.

METHODS

All-atom molecular dynamics simulations. The 50S subunit of the *E. Coli* ribosome (PDB ID 3R8T) was first oriented as previously described[1] and then cropped to form a rectangular box around the exit tunnel and 3 nm away from the PTC site. The final dimensions of the model are 8.5 x 10.4 x 9.6 (nm^3).

A simulation box was constructed with a minimum distance of 1 nm between the edge of the cropped ribosome and the periodic boundary wall in all dimensions. The system was solvated and then neutralized with Na^+ before adding 5 mM $MgCl_2$ and 100 mM NaCl. Next, the system was minimized with the steepest-decent algorithm to remove the bad contacts in the system. The system was allowed to equilibrate in the 1 ns of NVT ensemble and followed by 1 ns in the NPT ensemble. Harmonic potential restraints applied to all P and C_α atoms. We performed a production simulation in 20 ns with harmonic restraints applied to heavy atoms of the ribosome that were more than 30 Å from the x-axis to allow ribosome exit tunnel to reach equilibrium. All simulations were carried out with GROMACS 2018[2] using AMBER99SB force field[3] and the TIP3P water model[4], this combination was used because AMBER99SB is widely used in all-atom simulations of Ribosome[5–7], and TIP3P was used in the original work that produces AMBER99SB parameters. The particle mesh Ewald method[8] was used to calculate the long-range electrostatic interactions beyond 1.2 nm. Lennard-Jones interactions were calculated with a cut-off distance of 1.2 nm. The Nose-Hoover thermostat[9,10] and Parrinello-Rahman barostat[11] were employed to maintain the temperature and pressure at 310 K and 1 atm, respectively. The LINCS algorithm[12] was used to constrain all bonds involved hydrogen atom and the integration time step was set to 2 fs.

We then chose the representative conformation which has the widest exit tunnel opening by using the RMSD-based clustering with the threshold of 0.7 Å, the threshold was chosen such that the total number of clusters and the probability distribution of clusters are reasonable; in our case, the threshold of 0.7 Å gave the most reasonable result, we got 7 clusters with the probability of 70.36%, 18.69%, 6.75%, 1.95%, 1.2%, 0.75%, and 0.3%, respectively. We tested with other thresholds, for example from 0.8 to 1 Å, we got only 3 clusters, too little or with the threshold of 0.6 Å, we got 38 clusters and many clusters contain only 1 member.

Umbrella sampling simulations. The starting structure of the cropped ribosome used in umbrella sampling[13,14] was taken from the representative structure in the previous simulation, which has the widest exit tunnel opening to make sure there will be water molecules between methane and tunnel wall.

To maintain the shape of the ribosome we applied a harmonic restraint with a force constant of $10{,}000\ kJ/(mol \cdot nm^2)$ on all heavy ribosomal atoms during umbrella sampling simulations. Because the ribosome exit tunnel is bent and not a perfect cylinder, the y-z position of the tunnel as a function of x was computed by MOLEonline 2.0 web server[15]. The pathway outside of the tunnel is simply translated along a vector made by the last two points in the tunnel to maximize the distance between methane and ribosome surface. Positions A and B are approximately 7.6 nm and 6.0 nm away from the PTC, respectively (Fig. 1b in the main text). Position A is in the vestibule region, where the tertiary structure can be observed and position B is in the lower tunnel, where the helix formation is observed.

Holding one methane fixed at position A (or B), the other methane is brought along the center line (Fig. 1b in the main text), the reaction coordinate was equal to the x coordinate separation of the two methane molecules, along the center line, the distance between two consecutive windows was roughly 0.8 Å.

A harmonic potential was used to keep the methane molecule near the center of each window:

$$V_i = \frac{1}{2}k(\varsigma - \varsigma_i)^2. \qquad \text{(S1)}$$

With a force constant $k = 1000\ kJ/(mol \cdot nm^2)$, ς_i is the center of umbrella i. Another strong harmonic potential acting in YZ plane with force constant equal to $100{,}000\ kJ/(mol \cdot nm^2)$ was used to keep methane molecule in the center of the ribosome tunnel. For each umbrella, the simulation was equilibrated for 1ns of NVT and then 1ns of NPT followed by production run. The methane positions were collected every 100 fs for the well converged potential of mean force. There are 50 umbrellas used in the simulations involving position A, each umbrella is 190 ns, resulting 9.5 µs per PMF curve, and 72 umbrellas for the simulations involving position B, 60 ns for each umbrella, resulting 4.32 µs per PMF curve.

The methane molecule was parameterized using the General Amber Force Field[16] (GAFF) using the ACPYPE tool[17], the RESP[18] charges associated with the atoms of methane were calculated using GAUSSIAN09 package[19] with HF/6-31G* basic set.

The same pathway and protocol were used for umbrella sampling simulations in bulk solution, except without the presence of the ribosome.

The PMFs were calculated by using the weighted histogram analysis method[20,21]. The PMF at ς = 3.7 nm and 5.5 nm were chosen as a reference point (where all PMF profiles were defined to zero) for simulations at position A and position B, respectively.

For the simulation at position A, as mentioned above, we performed 190ns for each umbrella and used the last 160 ns for analyses. Trajectories were cut into 16 blocks of the block size 10-ns.

To calculate the entropy contribution to the PMF or ΔG, we performed two more sets of umbrella sampling at T = 300 K and 320 K and then utilize the finite difference temperature[22] of the ΔG at each inter solute separation r:

$$-T\Delta S(r) = T\frac{\Delta G(r, T + \Delta T) - \Delta G(r, T - \Delta T)}{2\Delta T} \qquad \text{(S2)}$$

Here, we used T = 310 K and ΔT = 10 K. The enthalpy component is:

$$\Delta H(r, T) = \Delta G(r, T) + T\Delta S(r) \qquad \text{(S3)}$$

For each block of data, we computed the differences in free energy, entropy, and enthalpy between the contact minimum and the solvent-separated minimum. The 95% confident intervals were calculated using the bootstrap sampling method with 10,000 iterations.

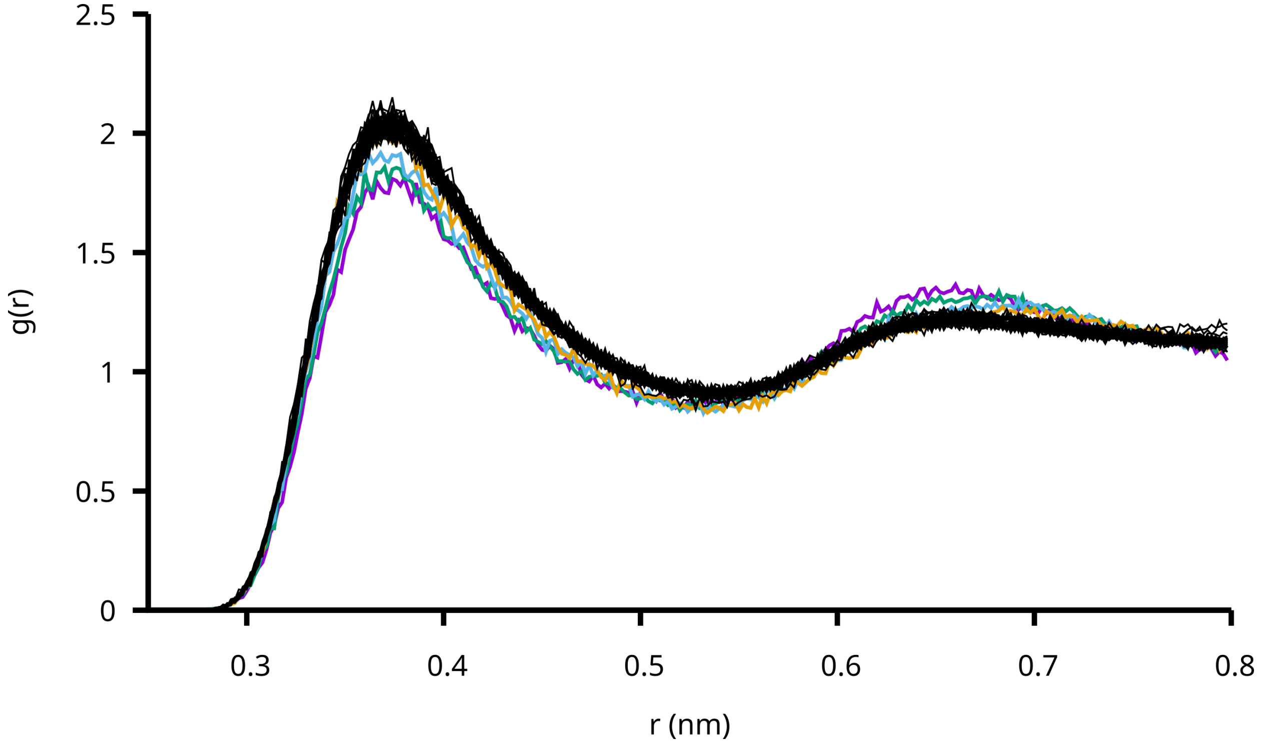

Figure S1. Radial distribution function g(r) of water Oxygen around the Carbon atom of the second methane (which moved along the center of the exit tunnel) in the simulation at position A. g(r) of the first four umbrella windows are plotted with color and the remained umbrella windows are plotted in black color. The water density at the first solvation shell around carbon methane in the first four umbrellas is lower than the other because in this case, two methanes were close to each other.

In general, few nanoseconds are sufficient to achieve convergence of association free energy of small molecules like methane in water[23,24]. In the ribosome exit tunnel, we need to sample longer for three reasons: (1) Diffusion of water molecules is slower[25], (2) the anisotropic environment means we do not have the advantages of degeneracy and symmetry found in bulk simulations to calculate partition functions, and (3) the sub kcal/mol differences in thermodynamic quantities requires much greater statistics to detect.

Hence, we sample up to 190 ns per umbrella along the exit tunnel, and the space between two umbrellas was small (0.8 Å), our uncertainty estimation and the statistical test showed that we have achieved a good sampling.

To test whether the difference of thermodynamic quantities: ΔΔG, TΔΔS, and ΔΔH (going from the solvent-separated minimum to the contact minimum) in the presence and absence of the ribosome are significant, we performed the one-tailed permutation test with 10^6 iterations.

The total simulation time required for getting significant results in the permutation test at position A is 57 μs (2 systems x 3 temperatures/ system x 9.5 μs / temperature).

In the simulations involving position B, we only calculated the potential of mean force and its uncertainty because of getting the convergence in entropy, enthalpy is much more expensive than PMF, and therefore, we employed the Bayesian bootstrapping of complete histograms method to estimate the uncertainty.

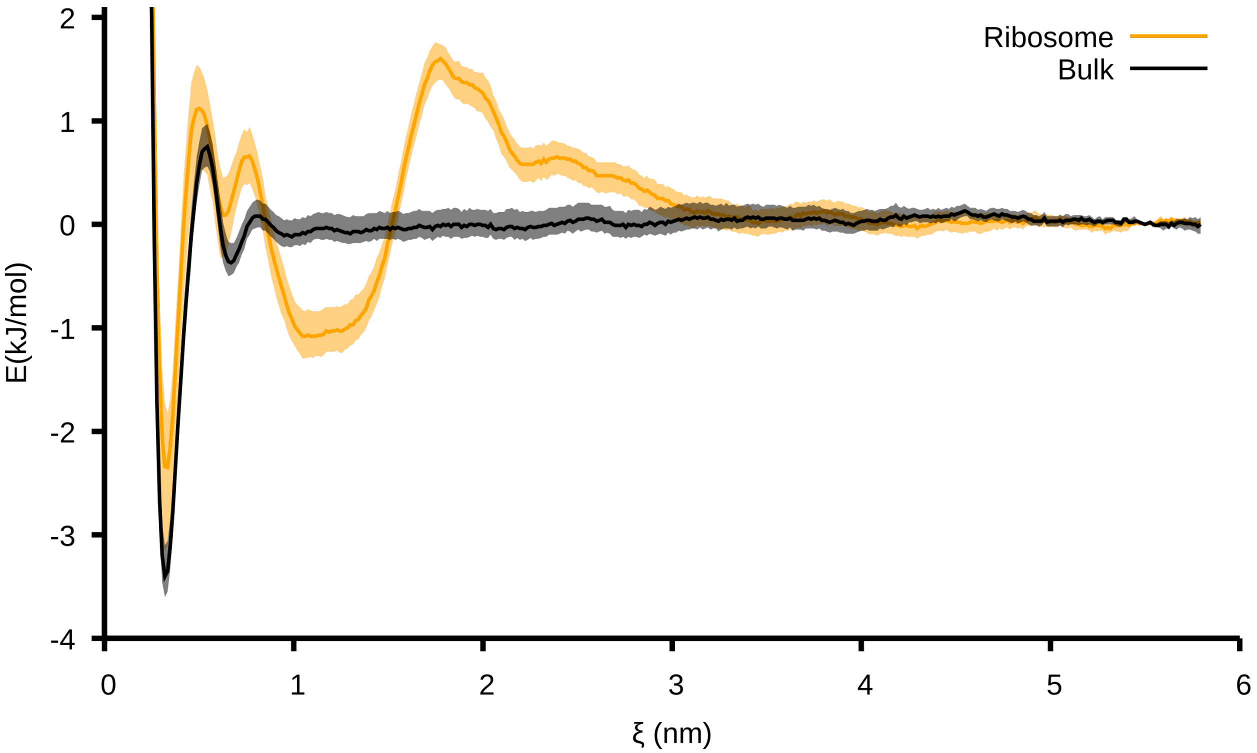

Figure S2. PMF of methane association in bulk (black) and in the Ribosome exit tunnel (orange) at position B. The shaded regions present 95% confident intervals calculated from Bayesian bootstrapping of complete histogram method.

Calculating the entropy of water. The Two-Phase Thermodynamic (2PT) method[26–28] was used to calculate the entropy of water. In this method, the density of state (DoS) functions (translation, rotation, and vibration motions) are determined from the Fourier transform of the corresponding velocity autocorrelation functions. The density of states of the system is partitioned into a gas-like and solid-like contribution, and then the thermodynamic properties of the system are calculated by weighting the individual density of states with the proper function. The theoretical details of the 2PT method have been previously reported[26–28]. The 2PT method has been applied to study the behavior of water in various systems[29–31] and been shown to efficiently compute entropies from short simulations (a time scale of 20 ps is sufficient for accurate thermodynamic properties).

The representative structure of the cropped ribosome in water was further equilibrated in NPT ensemble for 20 ns at 310 K and 1 atm. Next, three sets of 20 ps duration in NVT ensemble at 310 K were simulated and coordinates were collected every 4 fs with time step is set to 2 fs for the entropy calculations. In each trajectory, we selected a layer of water within 8 Å around position A, position B. Water molecules that resided continuously 15 Å away from the ribosome surface were considered as in the bulk region. We only considered water molecules that reside continuously in a given region for 20 ps and their thermodynamic properties were calculated. The 95% confident intervals were calculated using the bootstrap sampling method with 10,000 iterations.

Tetrahedral order parameters. The structural water ordering was detected by tetrahedral parameters[32,33]. The representative structure of cropped ribosome was further equilibrated in NPT ensemble for 20 ns. The tetrahedral parameters of water molecule were calculated from the production simulation in the NVT ensemble of 7 ns with a time step of 1 fs and coordinates were saved every 25 fs. We computed q and S_k at each point along the center line by selecting water molecules that were closest to the center line and its four nearest-neighbor water molecules. The oxygen positions of those water molecules will be used to calculate the order parameters as below.

Translational order parameter S_k:

$$S_k = 1 - \frac{1}{3}\sum_{k=1}^{4} \frac{(r_k - \bar{r})^2}{4\bar{r}^2} \tag{S4}$$

S_k measures the variance of the radial distances between central water oxygen and the four nearest neighbors water oxygen. Where r_k is the radial distance from the central oxygen atom to the kth peripheral oxygen atom and $\bar{r}$ is the mean value of four radial distances. S_k increases when the local tetrahedral order increases and reaches a maximum value of 1 for a perfect tetrahedron arrangement.

Orientational order parameter *q*:

The orientational order parameter measures how far the directions of the surrounding four nearest neighbors are from a tetrahedral arrangement. Here, we used the rescaled equation suggested by *Errington* and *Debenedetti*[34] :

$$q = 1 - \frac{3}{8}\sum_{j=1}^{3}\sum_{k=j+1}^{4}\left(\cos\psi_{jk} + \frac{1}{3}\right)^2 \tag{S5}$$

The rescaled version of q is defined in the way such that if the molecules are in random arrangement, then the six angles associated with the center molecules are independent, thus $\langle q \rangle = 0$. In the case of perfect tetrahedral network, $\cos\psi_{jk} = -\frac{1}{3}$, then $\langle q \rangle = 1$.

The 95% confidence intervals for q and S_k were calculated via bootstrap sampling method using 1,000 iterations.

References

1 D. A. Nissley, Q. V. Vu, F. Trovato, N. Ahmed, Y. Jiang, M. S. Li and E. P. O'Brien, *J. Am. Chem. Soc.*, 2020, **142**, 6103–6110.

2 M. J. Abraham, T. Murtola, R. Schulz, S. Páll, J. C. Smith, B. Hess and E. Lindah, *SoftwareX*, 2015, **1**–**2**, 19–25.

3 V. Hornak, R. Abel, A. Okur, B. Strockbine, A. Roitberg and C. Simmerling, *Proteins Struct. Funct. Genet.*, 2006, **65**, 712–725.

4 W. L. Jorgensen, J. Chandrasekhar, J. D. Madura, R. W. Impey and M. L. Klein, *J. Chem. Phys.*, 1983, **79**, 926–935.

5 L. V. Bock, C. Blau, G. F. Schröder, I. I. Davydov, N. Fischer, H. Stark, M. V. Rodnina, A. C. Vaiana and H. Grubmüller, *Nat. Struct. Mol. Biol.*, 2013, **20**, 1390–1396.

6 L. V. Bock, C. Blau, A. C. Vaiana and H. Grubmüller, *Nucleic Acids Res.*, 2015, **43**, 6747–6760.

7 S. Arenz, L. V. Bock, M. Graf, C. A. Innis, R. Beckmann, H. Grubmüller, A. C. Vaiana and D. N. Wilson, *Nat. Commun.*, , DOI:10.1038/ncomms12026.

8 T. Darden, D. York and L. Pedersen, *J. Chem. Phys.*, 1993, **98**, 10089–10092.

9 S. Nosé, *J. Chem. Phys.*, 1984, **81**, 511–519.

10 William G. Hoover, *Phys. Rev. A*, 1985, **31**, 1695–1697.

11 M. Parrinello and A. Rahman, *J. Appl. Phys.*, 1981, **52**, 7182–7190.

12 B. Hess, H. Bekker, H. J. C. Berendsen and J. G. E. M. Fraaije, *J. Comput. Chem.*, 1997, **18**, 1463–1472.

13 J. . Torrie, G. M Valleau, *J. Comput. Phys.*, 1977, **23**, 187.

14 J. Kästner, *Wiley Interdiscip. Rev. Comput. Mol. Sci.*, 2011, **1**, 932–942.

15 K. Berka, O. Hanák, D. Sehnal, P. Banáš, V. Navrátilová, D. Jaiswal, C. M. Ionescu, R. Svobodová Vařeková, J. Koča and M. Otyepka, *Nucleic Acids Res.*, 2012, **40**, 222–227.

16 J. Wang, R. M. Wolf, J. W. Caldwell, P. A. Kollman and D. A. Case, *J. Comput. Chem.*, 2004, **25**, 1157–1174.

17 A. W. Sousa Da Silva and W. F. Vranken, *BMC Res. Notes*, 2012, **5**, 1–8.

18 C. I. Bayly, P. Cieplak, W. D. Cornell and P. A. Kollman, *J. Phys. Chem.*, 1993, **97**, 10269–10280.

19 M. J. Frisch, G. W. Trucks, H. B. Schlegel, G. E. Scuseria, M. A. Robb, J. R. Cheeseman, G. Scalmani, V. Barone, B. Mennucci, G. A. Petersson, H. Nakatsuji, M. Caricato, X. Li, H. P. Hratchian, A. F. Izmaylov, J. Bloino, G. Zheng, J. L. Sonnenberg, M. Hada, M. Ehara, K. Toyota, R. Fukuda, J. Hasegawa, M. Ishida, T. Nakajima, Y. Honda, O. Kitao, H. Nakai, T. Vreven, J. A. Montgomery, J. E. Peralta, F. Ogliaro, M. Bearpark, J. J. Heyd, E. Brothers, K. N. Kudin, V. N. Staroverov, R. Kobayashi, J. Normand, K. Raghavachari, A. Rendell, J. C. Burant, S. S. Iyengar, J. Tomasi, M. Cossi, N. Rega, J. M. Millam, M. Klene, J. E. Knox, J. B. Cross, V. Bakken, C. Adamo, J. Jaramillo, R. Gomperts, R. E. Stratmann, O. Yazyev, A. J. Austin, R. Cammi, C. Pomelli, J. W. Ochterski, R. L. Martin,

K. Morokuma, V. G. Zakrzewski, G. A. Voth, P. Salvador, J. J. Dannenberg, S. Dapprich, A. D. Daniels, Ö. Farkas, J. B. Foresman, J. V Ortiz, J. Cioslowski and D. J. Fox, 2009.

20 S. Kumar, J. M. Rosenberg, D. Bouzida, R. H. Swendsen and P. A. Kollman, *J. Comput. Chem.*, 1992, **13**, 1011–1021.

21 J. S. Hub, B. L. De Groot and D. Van Der Spoel, *J. Chem. Theory Comput.*, 2010, **6**, 3713–3720.

22 N. Choudhury and B. Montgomery Pettitt, *J. Phys. Chem. B*, 2006, **110**, 8459–8463.

23 E. Sobolewski, M. Makowski, C. Czaplewski, A. Liwo, S. Ołdziej and H. A. Scheraga, *J. Phys. Chem. B*, 2007, **111**, 10765–10774.

24 S. Parui and B. Jana, *J. Phys. Chem. B*, 2017, **121**, 7016–7026.

25 D. Lucent, C. D. Snow, C. E. Aitken and V. S. Pande, *PLoS Comput. Biol.*, 2010, **6**, e1000963.

26 S. T. Lin, M. Blanco and W. A. Goddard, *J. Chem. Phys.*, 2003, **119**, 11792–11805.

27 S. T. Lin, P. K. Maiti and W. A. Goddard, *J. Phys. Chem. B*, 2010, **114**, 8191–8198.

28 T. A. Pascal, S. T. Lin and W. A. Goddard, *Phys. Chem. Chem. Phys.*, 2011, **13**, 169–181.

29 B. Jana, S. Pal, P. K. Maiti, S. T. Lin, J. T. Hynes and B. Bagchi, *J. Phys. Chem. B*, 2006, **110**, 19611–19618.

30 A. Debnath, B. Mukherjee, K. G. Ayappa, P. K. Maiti and S.-T. Lin, *J. Chem. Phys.*, 2010, **133**, 174704.

31 H. Kumar, B. Mukherjee, S.-T. Lin, C. Dasgupta, A. K. Sood and P. K. Maiti, *J. Chem. Phys.*, 2011, **134**, 124105.

32 P. Chau and A. J. Hardwick, *Mol. Phys.*, 1998, **93**, 511–518.

33 E. Duboué-Dijon and D. Laage, *J. Phys. Chem. B*, 2015, **119**, 8406–8418.

34 J. R. Errington and P. G. Debenedetti, *Nature*, 2001, **409**, 318–321.
